# Supplementary material for: Physical Activity in Community-Dwelling Older Adults: Which Real-World Accelerometry Measures Are Robust? A Systematic Review
Source: Sensors (Basel). 2023 Sep 2;23(17):7615. doi: 10.3390/s23177615 (PMC10490754; doi:10.3390/s23177615)
Supplement: Supplementary file 1 [file sensors-23-07615-s001.zip › sensors-2537739-supplementary.pdf]

Table S1

MEDLINE/PsycINFO/Embase/CINAHL/AMED {Conducted on 18 January 2021}

1. Fitness Trackers/
2. Accelerometry/ or Actigraphy/
3. Wearable Electronic Devices/
4. (fitness tracker* or acceleromet* or actigraph* or wearable* or smartwatch* or "smart watch*" or "activity track*" or step or pedomet*).mp. [mp=title, abstract, original title, name of substance word, subject heading word, floating sub-heading word, keyword heading word, organism supplementary concept word, protocol supplementary concept word, rare disease supplementary concept word, unique identifier, synonyms]
5. 1 or 2 or 3 or 4
6. exercise/ or exp running/ or swimming/ or exp walking/
7. motor activity/ or freezing reaction, cataleptic/
8. (exercis* or "motor activit*" or "physical activit*" or "habitual activit*" or "ambulatory activit*" or walk* or gait* or locomoti*).mp. [mp=title, abstract, original title, name of substance word, subject heading word, floating sub-heading word, keyword heading word, organism supplementary concept word, protocol supplementary concept word, rare disease supplementary concept word, unique identifier, synonyms]
9. 6 or 7 or 8
10. exp aged/ or "aged, 80 and over"/
11. ("older adult*" or "senior citizen*" or "older people" or elderly).mp. [mp=title, abstract, original title, name of substance word, subject heading word, floating sub-heading word, keyword heading word, organism supplementary concept word, protocol supplementary concept word, rare disease supplementary concept word, unique identifier, synonyms]
12. 10 or 11
13. exp "reproducibility of results"/ or sample size/ or exp "sensitivity and specificity"/
14. ("reproducibility of result*" or clinimetr* or variabilit* or reliab* or valid* or responsiveness or qualit* or specificity or sensitivity).mp. [mp=title, abstract, original title, name of substance word, subject heading word, floating sub-heading word, keyword heading word, organism supplementary concept word, protocol supplementary concept word, rare disease supplementary concept word, unique identifier, synonyms]
15. 13 or 14
16. 5 and 9 and 12 and 15
17. limit 16 to (english language and humans and yr="2010 - 2021")

WOS:

TOPIC:
("fitness tracker*" OR acceleromet* OR actigraph* OR wearable* OR smartwatch* OR "smart watch*" OR "activity track*" OR step OR pedomet*)
Databases= WOS, CCC, DRCI, DIIDW, KJD, MEDLINE, RSCI, SCIELO Timespan=2010-2020
Search language= English

TOPIC:
(exercis* OR "motor activit*" OR "physical activit*" OR "habitual activit*" OR "ambulatory activit*'" OR walk* OR gait* OR locomoti*)
Databases= WOS, CCC, DRCI, DIIDW, KJD, MEDLINE, RSCI, SCIELO Timespan=2010-2020
Search language= English

TOPIC:
("older adult*" OR "senior citizen*" OR "older people" OR elderly)
Databases= WOS, CCC, DRCI, DIIDW, KJD, MEDLINE, RSCI, SCIELO Timespan=2010-2020
Search language= English

TOPIC:
("reproducibility of result*" OR clinimetr* OR variabilit* OR reliab* OR valid* OR responsiveness OR qualit* OR specificity OR sensitivity)
Databases= WOS, CCC, DRCI, DIIDW, KJD, MEDLINE, RSCI, SCIELO Timespan=2010-2020
Search language=English

SCOPUS:

(TITLE-ABS-KEY (("reproducibility of result*" OR clinimetr* OR variabilit* OR reliab* OR valid* OR responsiveness OR qualit* OR specificity OR sensitivity))) AND ((TITLE-ABS-KEY ("fitness tracker*" OR acceleromet* OR actigraph* OR wearable* OR smartwatch* OR "smart watch*" OR "activity track*" OR step OR pedomet*)) AND (TITLE-ABS-KEY ((exercis* OR "motor activit*" OR "physical activit*" OR "habitual activit*" OR "ambulatory activit*'" OR walk* OR gait* OR locomoti*))) AND (TITLE-ABS-KEY (("older adult*" OR "senior citizen*" OR "older people" OR elderly)))) AND (PUBYEAR > 2009) AND (LIMIT-TO (LANGUAGE , "English"))

IEEE

(((("fitness tracker" OR acceleromet* OR actigraph OR actigraphy OR wearable OR smartwatch OR "smart watch" OR "activity tracker" OR step OR pedometer) AND (exercise OR "motor activit*" OR "physical activit*" OR "habitual activit*" OR "ambulatory activit*'" OR walk OR gait OR locomotion)))) AND ((("older adult" OR "senior citizen" OR "older people" OR elderly) AND ("reproducibility of result" OR "reproducibility of results" OR clinimetr* OR variability OR reliab* OR validity OR responsiveness OR quality OR specificity OR sensitivity)))
[Date range: 2010 - 2021]

MEDLINE/PsycINFO/Embase/CINAHL/AMED {Conducted on 25 November 2022}

1. Fitness Trackers/
2. Accelerometry/ or Actigraphy/
3. Wearable Electronic Devices/

4. (fitness tracker* or acceleromet* or actigraph* or wearable* or smartwatch* or "smart watch*" or "activity track*" or step or pedomet*).mp. [mp=title, abstract, original title, name of substance word, subject heading word, floating sub-heading word, keyword heading word, organism supplementary concept word, protocol supplementary concept word, rare disease supplementary concept word, unique identifier, synonyms]
5. 1 or 2 or 3 or 4
6. exercise/ or exp running/ or swimming/ or exp walking/
7. motor activity/ or freezing reaction, cataleptic/
8. (exercis* or "motor activit*" or "physical activit*" or "habitual activit*" or "ambulatory activit*" or walk* or gait* or locomoti*).mp. [mp=title, abstract, original title, name of substance word, subject heading word, floating sub-heading word, keyword heading word, organism supplementary concept word, protocol supplementary concept word, rare disease supplementary concept word, unique identifier, synonyms]
9. 6 or 7 or 8
10. exp aged/ or "aged, 80 and over"/
11. ("older adult*" or "senior citizen*" or "older people" or elderly).mp. [mp=title, abstract, original title, name of substance word, subject heading word, floating sub-heading word, keyword heading word, organism supplementary concept word, protocol supplementary concept word, rare disease supplementary concept word, unique identifier, synonyms]
12. 10 or 11
13. exp "reproducibility of results"/ or sample size/ or exp "sensitivity and specificity"/
14. ("reproducibility of result*" or clinimetr* or variabilit* or reliab* or valid* or responsiveness or qualit* or specificity or sensitivity).mp. [mp=title, abstract, original title, name of substance word, subject heading word, floating sub-heading word, keyword heading word, organism supplementary concept word, protocol supplementary concept word, rare disease supplementary concept word, unique identifier, synonyms]
15. 13 or 14
16. 5 and 9 and 12 and 15
17. limit 16 to (english language and humans and yr="2020 - 2022")

WOS:

TOPIC:
("fitness tracker*" OR acceleromet* OR actigraph* OR wearable* OR smartwatch* OR "smart watch*" OR "activity track*" OR step OR pedomet*)
Databases= WOS, CCC, DRCI, DIIDW, KJD, MEDLINE, RSCI, SCIELO Timespan=2020-2022
Search language= English

TOPIC:

(exercis* OR "motor activit*" OR "physical activit*" OR "habitual activit*" OR "ambulatory activit*'" OR walk* OR gait* OR locomoti*)
Databases= WOS, CCC, DRCI, DIIDW, KJD, MEDLINE, RSCI, SCIELO Timespan=2020-2022
Search language= English

TOPIC:
("older adult*" OR "senior citizen*" OR "older people" OR elderly)
Databases= WOS, CCC, DRCI, DIIDW, KJD, MEDLINE, RSCI, SCIELO Timespan=2020-2022
Search language= English

TOPIC:
("reproducibility of result*" OR clinimetr* OR variabilit* OR reliab* OR valid* OR responsiveness OR qualit* OR specificity OR sensitivity)
Databases= WOS, CCC, DRCI, DIIDW, KJD, MEDLINE, RSCI, SCIELO Timespan=2020-2022
Search language=English

SCOPUS:

(TITLE-ABS-KEY (("reproducibility of result*" OR clinimetr* OR variabilit* OR reliab* OR valid* OR responsiveness OR qualit* OR specificity OR sensitivity))) AND ((TITLE-ABS-KEY ("fitness tracker*" OR acceleromet* OR actigraph* OR wearable* OR smartwatch* OR "smart watch*" OR "activity track*" OR step OR pedomet*)) AND (TITLE-ABS-KEY ((exercis* OR "motor activit*" OR "physical activit*" OR "habitual activit*" OR "ambulatory activit*'" OR walk* OR gait* OR locomoti*))) AND (TITLE-ABS-KEY (("older adult*" OR "senior citizen*" OR "older people" OR elderly)))) AND (PUBYEAR > 2019) AND (LIMIT-TO (LANGUAGE , "English"))

IEEE

(((("fitness tracker" OR acceleromet* OR actigraph OR actigraphy OR wearable OR smartwatch OR "smart watch" OR "activity tracker" OR step OR pedometer) AND (exercise OR "motor activit*" OR "physical activit*" OR "habitual activit*" OR "ambulatory activit*'" OR walk OR gait OR locomotion)))) AND ((("older adult" OR "senior citizen" OR "older people" OR elderly) AND ("reproducibility of result" OR "reproducibility of results" OR clinimetr* OR variability OR reliab* OR validity OR responsiveness OR quality OR specificity OR sensitivity)))
[Date range: 2020 - 2022]

Supplementary Table S2. Acceptability and adherence of tools/instruments of studies included in the systematic review

Study	Instrument type	Placement of instrument	Length of wear	Sampling frequency (Hz)	Justification for location of wear	Adherence Level
Awais et al. [1]	tri-axial accelerometer, gyroscope and magnetometer	Chest, L5 and thigh (uSense); non-dominant wrist (Shimmer3)	Approximately 100 minutes	100	ND	ND
Brand et al. [2]	tri-axial accelerometer	Wrist	Up to 10 days	25 (Garmin), 100 (AX3)	Wrist-worn sensors are widely used in research studies and clinical trials to quantify movement.	ND
Briggs et al. [3]	tri-axial accelerometer	Garmin and ActiGraph – nondominant wrist ActiGraph – nondominant hip	Minimum of 48 hours	30 (ActiGraph), ND (Garmin)	Wrist-worn consumer accelerometry devices are more popular now.	ND
Burton et al. [4]	tri-axial accelerometer	Wrist	14 days	GENEactiv recorded movement in 30hz and collapsed into 60s epochs	ND	16 of the 30 fitness trackers for 14-day collection had some missing data.
Chigateri et al. [5]	tri-axial accelerometer	Lower back (L5)	Scripted tasks – Duration of scripted tasks. walking time (s) (20.3 ± 6.9), non-walking time (80.0 ± 25.1) Unscripted tasks – walking time (s) (115.4 ± 89.7), non-walking time (s) (500.4 ± 96.1)		Wearable sensors placed on the waist provide highest accuracy in predicting human body movements since they are closest to the body's centre of mass.	ND

* ND – not described.

Supplementary Table S2. Acceptability and adherence of tools/instruments of studies included in the systematic review—continued

Study	Instrument type	Placement of instrument	Length of wear	Sampling frequency (Hz)	Justification for location of wear	Adherence Level
Dijkstra et al. [6]	tri-axial accelerometer	lower back	30 minutes	100	ND	ND
Farina et al. [7]	tri-axial accelerometer	Fitbit Charge HR – dominant wrist<br>Misfit Shine – dominant wrist and waist<br>ActiGraph GT3X+ – dominant waist<br>NL2000i – above midpoint of dominant kneecap	7 days	ActiGraph recorded in 10s epochs, Fitbit Charge HR recorded in 60s epochs	ND	Device wear time (min/day): 843 ± 65. Nine participants had missing data from at least 1 activity monitor due to loss of device, error in set up and device repositioning. Four participants had evidence of at least one day of non-wear ActiGraph data, and 7 had at least one day of ActiGraph data less than 10 hrs. Three participants were excluded as they had less than 4 days valid ActiGraph data.
Geraedts et al. [8]	3D-MEMS accelerometer with barometric pressure sensor	Around the neck (worn as a necklace)	Standardized movement protocol: for the period the duration of the standardized activities<br>Free movement protocol: 30 minutes<br>User evaluation: 7 days	Accelerometry data – 50; Barometric data – 25	"Least" intrusive to the user; more suitable for daily wearing	Participants wore the sensor over 1 week and provided feedback on comfort, weight, size and usability via questionnaire – scored between 1 and 5 (1 meaning "Do not agree at all" and 5 meaning "Completely agree"). The average score on the user evaluation questionnaire was 4.4 (SD ± 0.6; range 2.4–5.0) on a scale of 1 to 5.<br>All participants wore the sensor during daytime hours of all requested days, 16 (i.e., 80%) wore it while sleeping.

* ND – not described.

Supplementary Table S2. Acceptability and adherence of tools/instruments of studies included in the systematic review - continued

Study	Instrument type	Placement of instrument	Length of wear	Sampling frequency (Hz)	Justification for location of wear	Adherence Level
Kastelic et al. [9]	tri-axial accelerometer	Polar Vantage M (wrist); Garmin Vivoactive 4s (wrist); Garmin Vivosport (wrist); ActiGraph wGT3X-BT (waist)	Group baseline protocol – 6 days; Group extended protocol – 12 days (each wearable over 4 days)	ActiGraph GT3X+ – 30; Polar Vantage M (ND); Garmin Vivoactive 4s (ND); Garmin Vivosport (ND)	ND	The wear time compliance with the Polar Vantage M, Garmin Vivoactive 4s and Garmin Vivosport was as high as 24.0 ± 0.1 h/day, 23.9 ± 0.5 h/day and 23.9 ± 0.5 h/day, respectively
Paul et al. [10]	tri-axial accelerometer	Right hip	7 days	ActiGraph GT3X+ – 30	ND	ND
Soltani et al. [11]	tri-axial accelerometer	GENEactiv Original (wrist), ActiGraph GT9X (shank)	Around 12 hours (within one day)	GENEactiv Original – 40 ,ActiGraph GT9X – 50	It offers comfort, high usability and discreet monitoring thus, increasing user compliance.	ND
Taylor et al. [12]	tri-axial accelerometer	Lower back	Scripted – 4 to 6 minutes. Unscripted – 5 to 9 minutes	100	ND	ND
Yamada et al. [13]	Actimarker – tri-axial accelerometer; Lifecorder – uniaxial accelerometer	Back of the waist	14 days	ND	ND	ND

* ND – not described.

1. Awais, M.; Chiari, L.; Ihlen, E.A.; Helbostad, J.L.; Palmerini, L. Classical machine learning versus deep learning for the older adults free-living activity classification. *Sensors* **2021**, *21*, 4669.
2. Brand, Y.E.; Schwartz, D.; Gazit, E.; Buchman, A.S.; Gilad-Bachrach, R.; Hausdorff, J.M. Gait Detection from a Wrist-Worn Sensor Using Machine Learning Methods: A Daily Living Study in Older Adults and People with Parkinson's Disease. *Sensors* **2022**, *22*, 7094.
3. Briggs, B.C.; Hall, K.S.; Jain, C.; Macrea, M.; Morey, M.C.; Oursler, K.K. Assessing Moderate to Vigorous Physical Activity in Older Adults: Validity of a Commercial Activity Tracker. *Frontiers in Sports and Active Living* **2022**, *3*, 766317.
4. Burton, E.; Hill, K.D.; Lautenschlager, N.T.; Thogersen-Ntoumani, C.; Lewin, G.; Boyle, E.; Howie, E. Reliability and validity of two fitness tracker devices in the laboratory and home environment for older community-dwelling people. *BMC Geriatr* **2018**, *18*, 103, doi:10.1186/s12877-018-0793-4.
5. Chigateri, N.G.; Kerse, N.; Wheeler, L.; MacDonald, B.; Klenk, J. Validation of an accelerometer for measurement of activity in frail older people. *Gait Posture* **2018**, *66*, 114-117, doi:10.1016/j.gaitpost.2018.08.024.

6. Dijkstra, B.; Kamsma, Y.; Zijlstra, W. Detection of gait and postures using a miniaturised triaxial accelerometer-based system: accuracy in community-dwelling older adults. *Age and ageing* **2010**, *39*, 259-262.
7. Farina, N.; Lowry, R.G. The Validity of Consumer-Level Activity Monitors in Healthy Older Adults in Free-Living Conditions. *J Aging Phys Act* **2018**, *26*, 128-135, doi:10.1123/japa.2016-0344.
8. Geraedts, H.A.; Zijlstra, W.; Van Keeken, H.G.; Zhang, W.; Stevens, M. Validation and User Evaluation of a Sensor-Based Method for Detecting Mobility-Related Activities in Older Adults. *PLoS One* **2015**, *10*, e0137668, doi:10.1371/journal.pone.0137668.
9. Kastelic, K.; Dobnik, M.; Löfler, S.; Hofer, C.; Šarabon, N. Validity, reliability and sensitivity to change of three consumer-grade activity trackers in controlled and free-living conditions among older adults. *Sensors* **2021**, *21*, 6245.
10. Paul, S.S.; Tiedemann, A.; Hassett, L.M.; Ramsay, E.; Kirkham, C.; Chagpar, S.; Sherrington, C. Validity of the Fitbit activity tracker for measuring steps in community-dwelling older adults. *BMJ Open Sport Exerc Med* **2015**, *1*, e000013, doi:10.1136/bmjsem-2015-000013.
11. Soltani, A.; Paraschiv-Ionescu, A.; Dejnabadi, H.; Marques-Vidal, P.; Aminian, K. Real-world gait bout detection using a wrist sensor: An unsupervised real-life validation. *Ieee Access* **2020**, *8*, 102883-102896.
12. Taylor, L.M.; Klenk, J.; Maney, A.J.; Kerse, N.; MacDonald, B.M.; Maddison, R. Validation of a body-worn accelerometer to measure activity patterns in octogenarians. *Archives of physical medicine and rehabilitation* **2014**, *95*, 930-934.
13. Yamada, Y.; Hashii-Arishima, Y.; Yokoyama, K.; Itoi, A.; Adachi, T.; Kimura, M. Validity of a triaxial accelerometer and simplified physical activity record in older adults aged 64-96 years: a doubly labeled water study. *Eur J Appl Physiol* **2018**, *118*, 2133-2146, doi:10.1007/s00421-018-3944-6.
